# *N*,*N*′-Bis(pyridin-3-yl)oxamide

**DOI:** 10.1107/S1600536813007277

**Published:** 2013-03-23

**Authors:** Shih-Miao Liu, Hsiu-Yi He, Jhy-Der Chen

**Affiliations:** a Center for General Education, Hsin Sheng Junior College of Medical Care and Management, Longtan, Taiwan; b Department of Chemistry, Chung-Yuan Christian University, Chung-Li, Taiwan

## Abstract

The title mol­ecule, C_12_H_10_N_4_O_2_, located about an inversion centre, is roughly planar, with an r.m.s. deviation from the least-squares plane of all non-H atoms of 0.019 Å. In the crystal, N—H⋯N hydrogen bonds between the amide N—H group and the pyridine N atom connect the mol­ecules into a corrugated layer parallel to (10-1).

## Related literature

For *N,N*’-di(3-pyrid­yl)oxamide and its metal complexes, see: Hu *et al.* (2012 ▶).
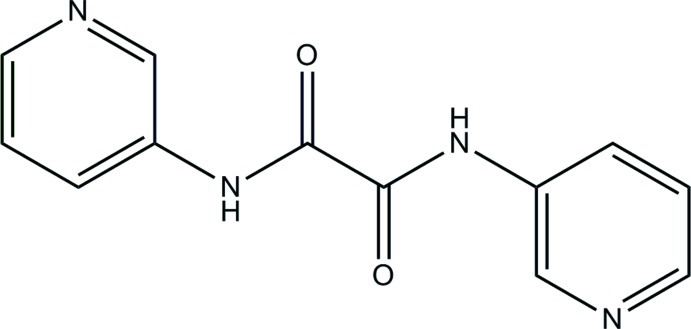


## Experimental

### 

#### Crystal data

C_12_H_10_N_4_O_2_
*M* *_r_* = 242.24
Monoclinic, 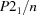

*a* = 3.8992 (7) Å
*b* = 12.662 (2) Å
*c* = 10.9678 (17) Å
β = 97.983 (4)°
*V* = 536.26 (16) Å^3^
*Z* = 2
Mo *K*α radiation
μ = 0.11 mm^−1^
*T* = 297 K
0.58 × 0.20 × 0.06 mm

#### Data collection

Bruker SMART 1000 diffractometer
Absorption correction: multi-scan (*SADABS*; Bruker, 1997 ▶) *T*_min_ = 1.000, *T*_max_ = 1.000
2997 measured reflections
1050 independent reflections
768 reflections with *I* > 2σ(*I*)
*R*_int_ = 0.034

#### Refinement

*R*[*F* ^2^ > 2σ(*F* ^2^)] = 0.043
*wR*(*F* ^2^) = 0.126
*S* = 1.06
1050 reflections
82 parameters
H-atom parameters constrained
Δρ_max_ = 0.19 e Å^−3^
Δρ_min_ = −0.26 e Å^−3^

### 

Data collection: *SMART* (Bruker, 1997 ▶); cell refinement: *SAINT* (Bruker, 1997 ▶); data reduction: *SAINT* and *SHELXTL* (Sheldrick, 2008 ▶); program(s) used to solve structure: *SHELXS97* (Sheldrick, 2008 ▶); program(s) used to refine structure: *SHELXL97* (Sheldrick, 2008 ▶); molecular graphics: *SHELXTL*; software used to prepare material for publication: *SHELXTL*.

## Supplementary Material

Crystal structure: contains datablock(s) I, global. DOI: 10.1107/S1600536813007277/gk2563sup1.cif

Structure factors: contains datablock(s) I. DOI: 10.1107/S1600536813007277/gk2563Isup2.hkl

Supplementary material file. DOI: 10.1107/S1600536813007277/gk2563Isup3.cml

Additional supplementary materials:  crystallographic information; 3D view; checkCIF report

## Figures and Tables

**Table 1:** Hydrogen-bond geometry (Å, °)

*D*—H⋯*A*	*D*—H	H⋯*A*	*D*⋯*A*	*D*—H⋯*A*
N1—H1*A*⋯N2^i^	0.86	2.26	3.061 (2)	156

Symmetry code: (i) 
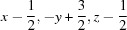
.

**Fig. 1.**
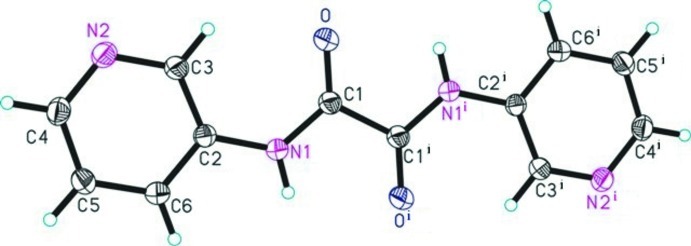
Molecular structure of the title compound with atom labeling and displacement ellipsoids drawn at the 30% probability level.

**Fig. 2.**
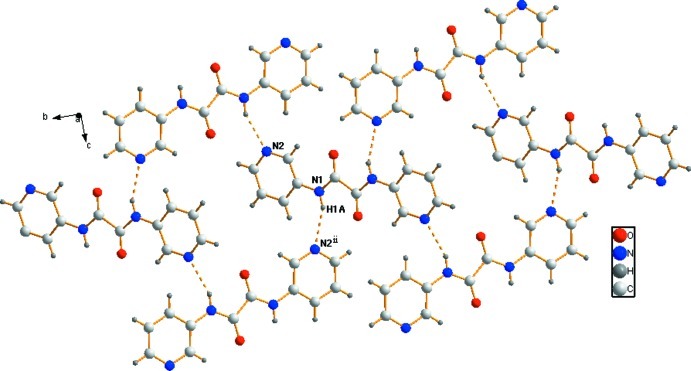
Hydrogen bonding interactions in the title compound.

## supplementary crystallographic information

### Comment

Several Zn(II), Cd(II) and Hg(II) complexes containing *N,N*'-di(3-pyridyl)oxamide ligands have been reported, which show one-dimensional chains and metallocycles (Hu *et al.*, 2012). Within this project the crystal structure of the title compound was determined (Fig. 1). In its crystal structure intermolecular N—H···N hydrogen bonds are found (Table 1 & Fig. 2).

### Experimental

The title compound was prepared according to a published procedure (Hu *et al.*, 2012). Block crystals suitable for X-ray crystallography were obtained by slow evaporation of the solvent from a solution of the title compound in methanol.

### Refinement

H atoms bound to C and N atoms were placed in idealized positions and constrained to ride on their parent atoms, with C—H = 0.93 Å and N—H = 0.86 Å, and with *U*_iso_(H) = 1.2 or 1.5 *U*_eq_(C/N).

### Crystal data

**Table:**

C_12_H_10_N_4_O_2_	*F*(000) = 252
*M**_r_* = 242.24	*D*_x_ = 1.500 Mg m^−^^3^
Monoclinic, *P*2_1_/*n*	Mo *K*α radiation, λ = 0.71073 Å
Hall symbol: -P 2yn	Cell parameters from 1108 reflections
*a* = 3.8992 (7) Å	θ = 2.5–25.6°
*b* = 12.662 (2) Å	µ = 0.11 mm^−^^1^
*c* = 10.9678 (17) Å	*T* = 297 K
β = 97.983 (4)°	Parallelepiped, colorless
*V* = 536.26 (16) Å^3^	0.58 × 0.20 × 0.06 mm
*Z* = 2	

### Data collection

**Table:**

Bruker SMART 1000 diffractometer	1050 independent reflections
Radiation source: fine-focus sealed tube	768 reflections with *I* > 2σ(*I*)
Graphite monochromator	*R*_int_ = 0.034
φ and ω scans	θ_max_ = 26.0°, θ_min_ = 2.5°
Absorption correction: multi-scan (*SADABS*; Bruker, 1997)	*h* = −4→4
*T*_min_ = 1.000, *T*_max_ = 1.000	*k* = −15→14
2997 measured reflections	*l* = −12→13

### Refinement

**Table:**

Refinement on *F*^2^	Primary atom site location: structure-invariant direct methods
Least-squares matrix: full	Secondary atom site location: difference Fourier map
*R*[*F*^2^ > 2σ(*F*^2^)] = 0.043	Hydrogen site location: inferred from neighbouring sites
*wR*(*F*^2^) = 0.126	H-atom parameters constrained
*S* = 1.06	*w* = 1/[σ^2^(*F*_o_^2^) + (0.075*P*)^2^] where *P* = (*F*_o_^2^ + 2*F*_c_^2^)/3
1050 reflections	(Δ/σ)_max_ < 0.001
82 parameters	Δρ_max_ = 0.19 e Å^−^^3^
0 restraints	Δρ_min_ = −0.26 e Å^−^^3^

### Special details

**Table:**

Geometry. All e.s.d.'s (except the e.s.d. in the dihedral angle between two l.s. planes) are estimated using the full covariance matrix. The cell e.s.d.'s are taken into account individually in the estimation of e.s.d.'s in distances, angles and torsion angles; correlations between e.s.d.'s in cell parameters are only used when they are defined by crystal symmetry. An approximate (isotropic) treatment of cell e.s.d.'s is used for estimating e.s.d.'s involving l.s. planes.
Refinement. Refinement of *F*^2^ against ALL reflections. The weighted *R*-factor *wR* and goodness of fit *S* are based on *F*^2^, conventional *R*-factors *R* are based on *F*, with *F* set to zero for negative *F*^2^. The threshold expression of *F*^2^ > σ(*F*^2^) is used only for calculating *R*-factors(gt) *etc*. and is not relevant to the choice of reflections for refinement. *R*-factors based on *F*^2^ are statistically about twice as large as those based on *F*, and *R*- factors based on ALL data will be even larger.

### Fractional atomic coordinates and isotropic or equivalent isotropic displacement parameters (Å^2^)

**Table:**

	*x*	*y*	*z*	*U*_iso_*/*U*_eq_	
O	0.1096 (4)	0.96926 (10)	1.15577 (12)	0.0530 (5)	
N1	0.2264 (4)	0.87848 (10)	0.98550 (13)	0.0342 (4)	
H1A	0.2033	0.8827	0.9065	0.041*	
N2	0.6079 (4)	0.67892 (12)	1.21174 (13)	0.0421 (5)	
C1	0.0929 (5)	0.95887 (12)	1.04473 (16)	0.0344 (4)	
C2	0.3993 (4)	0.78835 (13)	1.03865 (15)	0.0309 (4)	
C3	0.4530 (5)	0.76739 (14)	1.16394 (16)	0.0382 (5)	
H3A	0.3784	0.8168	1.2173	0.046*	
C4	0.7184 (5)	0.60928 (14)	1.13512 (17)	0.0416 (5)	
H4A	0.8238	0.5476	1.1676	0.050*	
C5	0.6832 (5)	0.62458 (14)	1.00998 (17)	0.0404 (5)	
H5A	0.7667	0.5747	0.9593	0.048*	
C6	0.5223 (5)	0.71506 (13)	0.96066 (16)	0.0367 (5)	
H6A	0.4964	0.7269	0.8762	0.044*	

### Atomic displacement parameters (Å^2^)

**Table:**

	*U*^11^	*U*^22^	*U*^33^	*U*^12^	*U*^13^	*U*^23^
O	0.0796 (11)	0.0451 (9)	0.0339 (8)	0.0183 (7)	0.0064 (7)	−0.0030 (6)
N1	0.0433 (9)	0.0310 (8)	0.0283 (8)	0.0024 (6)	0.0057 (6)	0.0002 (6)
N2	0.0547 (11)	0.0349 (9)	0.0351 (9)	−0.0016 (7)	0.0012 (7)	0.0017 (7)
C1	0.0387 (10)	0.0312 (9)	0.0337 (9)	−0.0020 (7)	0.0065 (7)	−0.0017 (7)
C2	0.0339 (9)	0.0277 (8)	0.0313 (9)	−0.0046 (7)	0.0048 (7)	−0.0007 (7)
C3	0.0501 (12)	0.0311 (9)	0.0335 (10)	−0.0017 (7)	0.0066 (8)	−0.0028 (8)
C4	0.0479 (11)	0.0324 (9)	0.0428 (11)	0.0021 (8)	0.0001 (8)	0.0030 (8)
C5	0.0446 (11)	0.0354 (10)	0.0411 (10)	0.0039 (8)	0.0061 (8)	−0.0042 (8)
C6	0.0416 (11)	0.0380 (10)	0.0305 (9)	0.0014 (8)	0.0050 (7)	−0.0009 (8)

### Geometric parameters (Å, º)

**Table:**

O—C1	1.218 (2)	C2—C6	1.392 (2)
N1—C1	1.350 (2)	C3—H3A	0.9300
N1—C2	1.410 (2)	C4—C5	1.374 (3)
N1—H1A	0.8600	C4—H4A	0.9300
N2—C4	1.330 (2)	C5—C6	1.380 (2)
N2—C3	1.344 (2)	C5—H5A	0.9300
C1—C1^i^	1.541 (3)	C6—H6A	0.9300
C2—C3	1.387 (2)		
			
C1—N1—C2	127.27 (14)	N2—C3—H3A	118.5
C1—N1—H1A	116.4	C2—C3—H3A	118.5
C2—N1—H1A	116.4	N2—C4—C5	122.78 (17)
C4—N2—C3	118.26 (15)	N2—C4—H4A	118.6
O—C1—N1	126.31 (16)	C5—C4—H4A	118.6
O—C1—C1^i^	121.25 (19)	C4—C5—C6	119.04 (17)
N1—C1—C1^i^	112.44 (17)	C4—C5—H5A	120.5
C3—C2—C6	117.65 (16)	C6—C5—H5A	120.5
C3—C2—N1	124.21 (15)	C5—C6—C2	119.30 (16)
C6—C2—N1	118.14 (14)	C5—C6—H6A	120.3
N2—C3—C2	122.94 (16)	C2—C6—H6A	120.3

Symmetry code: (i) −*x*, −*y*+2, −*z*+2.

### Hydrogen-bond geometry (Å, º)

**Table:**

*D*—H···*A*	*D*—H	H···*A*	*D*···*A*	*D*—H···*A*
N1—H1*A*···N2^ii^	0.86	2.26	3.061 (2)	156

Symmetry code: (ii) *x*−1/2, −*y*+3/2, *z*−1/2.
